# Patient reported experiences of Swedish patients being investigated for cancer during the Covid-19 pandemic

**DOI:** 10.1007/s00520-023-07897-y

**Published:** 2023-06-24

**Authors:** Helena Tufvesson Stiller, Marcus Schmitt-Egenolf, Helena Fohlin, Srinivas Uppugunduri

**Affiliations:** 1 Department of Public Health and Clinical Medicine, Umeå University, Umeå, Sweden; 2 Regional Cancer Center Southeast, Linköping, Sweden; 3 Department of Biomedical and Clinical Sciences, Linköping University, Linköping, Sweden

**Keywords:** Cancer, Covid-19, Emotional support, Oncology, Patient reported experience measures, Patient satisfaction

## Abstract

**Background:**

Patient reported experiences in individuals being investigated for cancer have been recorded in a nationwide survey in Sweden, providing an opportunity to assess the impact of the Covid-19-pandemic.

**Material and Methods:**

Questionnaires from 45920 patients were analyzed to assess the experience of being investigated for cancer. Data from before the Covid-19-pandemic (2018–2019) was compared to data acquired during the pandemic (2020–2021), using chi-square and Wilcoxon rank sum tests. Both, patients who were cleared from suspicion of cancer and those who were diagnosed with cancer were included.

**Results:**

Fewer patients in total visited health services during the pandemic. However, patients that did seek help did so to a similar extent during as prior to the pandemic. Patient waiting time was perceived to be shorter during the pandemic and judged as neither too long nor too short by most patients. The emotional support to patients improved during the pandemic, whereas the support to next of kin declined. A majority of patients received the results from the investigation in a meeting with the physician. Although there was a preference for receiving results in a meeting with the physician, the pandemic has brought an increasing interest in receiving results by phone.

**Conclusion:**

Swedish cancer healthcare has shown resilience during the Covid-19-pandemic, maintaining high patient satisfaction while working under conditions of extraordinary pressure. Patients became more open to alternatives to physical “in person” health care visits which could lead to more digital visits in the future. However, support to significant others demands special attention.

## Background

The Covid-19 pandemic hit Sweden in early 2020 and had far reaching effects on the health care system in Sweden as well as globally. As in many other countries, resources were relocated from routine care to care for Covid-19 patients. Some screening programmes for cancer were put on hold and non-essential procedures were postponed [1]. Multiple studies have addressed the challenges of providing care to cancer patients during the pandemic as this group is both in high need of medical care while, at the same time, being more susceptible to higher mortality from Covid-19 [2–4]. As in other countries, the total number of tumors diagnosed in Sweden decreased significantly from 2019 to 2020, with the highest impact on prostate cancer [5], cervical cancer in situ and breast cancer [6]. Studies on patient experiences of cancer care during the Covid-19 pandemic are few. Reports from USA [7], Canada [8] and the Netherlands [9] show that patients had, to a varying degree, their visits cancelled or converted to digital meetings. Although some had no objections to this conversion to digital visits, others experienced stress [10, 11].

Swedish cancer care has for many years held high medical quality including high survival rates, despite long waiting times [12, 13]. However, the knowledge regarding patient experiences was limited. Consequently, the Swedish government embarked on an ambitious program in 2015 to reform cancer care and implemented the so-called *standardized cancer care pathways* based on the Danish model. The main purpose of the *standardized cancer care pathways* was to reduce the waiting time from the first suspicion of cancer to the start of treatment [14], as well as monitoring patient satisfaction with the investigations. A questionnaire was developed and used in the evaluation.

The objective of this study was to evaluate if patient satisfaction, the patterns for seeking care and the emotional support to patients and significant others have changed during the Covid-19 pandemic.

## Patients and methods

### Study design

Regional Cancer Centers and Swedish Association of Local Authorities and Regions collaborated to perform a longitudinal survey to monitor patient satisfaction with *standardized cancer care pathways* in the Swedish national cancer care system. An invitation to answer a web survey was sent by regular mail to patients that had been investigated for suspicion of cancer 6–10 weeks earlier. A reminder and a paper questionnaire were mailed to the patients if no response was recorded after 3 weeks. Our study, analyzing these data, was approved by the Swedish Ethical Review Authority, Gothenburg sub-committee (approval no 2021–04508) and is in accordance with the Declaration of Helsinki.

### Patient population

Patients who had been investigated for suspected cancer received an invitation. Both, patients that were freed from suspicion of having a cancer diagnosis and patients that received a cancer diagnosis were included. Patients from 8 out of 21 health care regions in Sweden were included in the study. These regions include both larger cities as well as rural areas and were chosen to obtain a comprehensive data set for our time periods of interest. The excluded regions lacked, due to the pandemic, data for 2–6 months during 2020. Patients had been investigated for one of the following: acute leukemia; anal cancer; brain tumor; breast cancer; cervical cancer; colorectal cancer; cutaneous melanoma; gall bladder cancer; gastroenteropancreatic neuroendocrine tumor; head- and neck cancer; kidney cancer; liver cancer; lung cancer; malignant lymphoma or CLL; myeloma; esophagus- and ventricular cancer; ovarian cancer; pancreatic cancer; penile cancer; prostate cancer; skeletal- and soft tissue sarcoma; testicular cancer; thyroid cancer; urothelial cancer; uterine cancer; visceral- and retroperitoneal sarcoma; vulvar cancer; cancer with unknown primary tumor or serious unspecific symptoms. For logistical reasons, a maximum of 50 patients per diagnosis and region were included each month. For most diagnoses this meant inclusion of all patients, but for larger diagnoses a random selection was made from all eligible patients. Patients under 18 years of age were excluded. In total 80 118 patients were invited to participate.

### Questionnaire

The questionnaire contained 33 fixed response questions and covered the dimensions overall impression, emotional support, involvement, respect, continuity, coordination, information, knowledge, accessibility as well as demographic information about the patient. In addition, a free text box was available for comments. The questions were based on previously used questionnaires and designed in collaboration with patient representatives and health care professionals. The questionnaire was tested on two separated focus groups for validation and adjusted according to the results of the discussions prior to the start of the survey. In this study, analysis was restricted to fixed responses only.

### Variables and statistics

The data was divided into two samples, ‘before Covid’ (January 2018-December 2019) and ‘Covid’ (April 2020-March 2021). The Chi-square test was used to compare the distributions of answers between the samples”before covid” and”covid”. The two-sided Wilcoxon rank sum test was used for ordered categorical variables. In case of a statistically significant result of the test for one of the questions, the chi-square test was used to compare the proportions between”before covid” and”covid” within each possible answer. In order to account for multiple testing, a p-value of < 0.01 was considered statistically significant for all tests. All statistical analyses were performed using R version 4.0.3. (R Core Team, Vienna, Austria). Missing data was handled by pairwise deletion.

## Results

A total of 45 920 patients consented to participate by sending in the questionnaire, giving a response rate of 57.3%. The patient population ‘before Covid’ was comparable to the ‘Covid’ population in terms of age, sex, type of cancer and whether they had received a cancer diagnosis or not (Table 1).

**Table 1:** Background parameters for the patient population before and during Covid-19

	Time period		
	Before Covid N (%)	Covid N (%)	Total N (%)
Sex			
Male	15544 (51.26)	7772 (49.83)	23316 (50.78)
Female	14779 (48.74)	7825 (50.17)	22604 (49.22)
Age			
18 – 29	281 (0.93)	200 (1.28)	481 (1.05)
30 – 49	2587 (8.53)	1577 (10.11)	4164 (9.07)
50 – 69	12298 (40.56)	6407 (41.08)	18705 (40.73)
70 +	15157 (49.99)	7413 (47.53)	22570 (49.15)
Cancer diagnosis			
No	17161 (56.59)	8568 (54.93)	25729 (56.03)
Yes	13162 (43.41)	7029 (45.07)	20191 (43.97)
Cancer Care Pathway			
Acute leukemia	73 (0.24)	34 (0.22)	107 (0.23)
Anal cancer	94 (0.31)	80 (0.51)	174 (0.38)
Brain tumor	221 (0.73)	104 (0.67)	325 (0.71)
Breast cancer	3976 (13.11)	2040 (13.08)	6016 (13.10)
Cervical cancer	102 (0.34)	135 (0.87)	237 (0.52)
Colorectal cancer	4385 (14.46)	1880 (12.05)	6265 (13.64)
Cutaneous melanoma	3182 (10.49)	1567 (10.05)	4749 (10.34)
Gall bladder cancer	163 (0.54)	67 (0.43)	230 (0.50)
GEP/NET	11 (0.04)	41 (0.26)	52 (0.11)
Head- and neck cancer	1820 (6.00)	863 (5.53)	2683 (5.84)
Kidney cancer	336 (1.11)	292 (1.87)	628 (1.37)
Liver cancer	240 (0.79)	95 (0.61)	335 (0.73)
Lung cancer	1758 (5.80)	939 (6.02)	2697 (5.87)
Malignant lymphoma or CLL	995 (3.28)	570 (3.65)	1565 (3.41)
Myeloma	450 (1.48)	207 (1.33)	657 (1.43)
Esophagus- and ventricular cancer	370 (1.22)	226 (1.45)	596 (1.30)
Ovarian cancer	587 (1.94)	225 (1.44)	812 (1.77)
Pancreatic cancer	577 (1.90)	251 (1.61)	828 (1.80)
Penile cancer	103 (0.34)	76 (0.49)	179 (0.39)
Prostate cancer	4686 (15.45)	2193 (14.06)	6879 (14.98)
Skeletal- and soft tissue sarcoma	112 (0.37)	193 (1.24)	305 (0.66)
Testicular cancer	114 (0.38)	153 (0.98)	267 (0.58)
Thyroid cancer	153 (0.50)	167 (1.07)	320 (0.70)
Urothelial cancer	4111 (13.56)	1677 (10.75)	5788 (12.60)
Uterine cancer	641 (2.11)	861 (5.52)	1502 (3.27)
Visceral- and retroperitoneal sarcoma	32 (0.11)	81 (0.52)	113 (0.25)
Vulvar cancer	35 (0.12)	60 (0.38)	95 (0.21)
Cancer with unknown primary tumor	251 (0.83)	142 (0.91)	393 (0.86)
Serious unspecific symptoms	745 (2.46)	378 (2.42)	1123 (2.45)
Total	30323	15597	45920

*CLL* chronic lymphocytic leukemia; *GEP/NET* gastroenteropancreatic neuroendocrine tumor

**Fig. 1:**
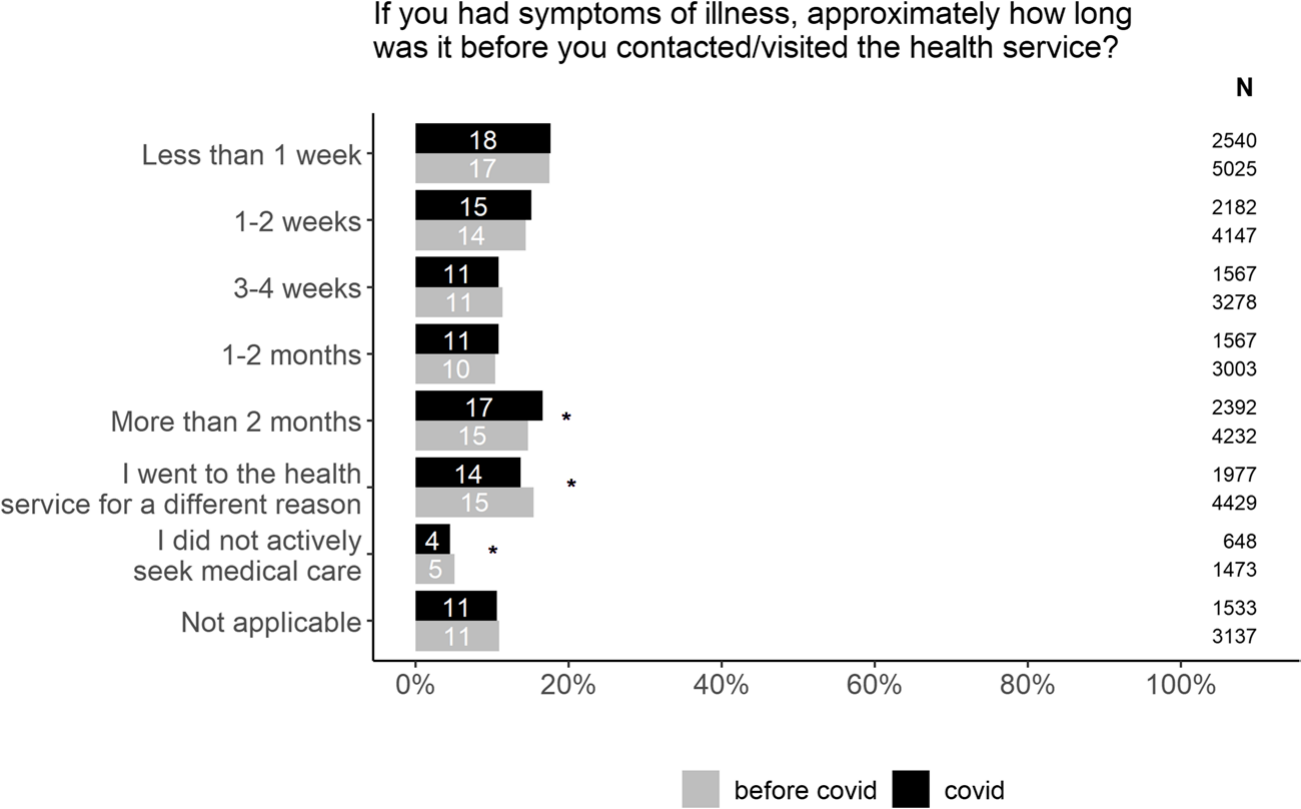
Patient delay before and during the Covid-19 pandemic. The chi-square test was used to compare the proportions between”before covid” and”during covid” within each possible answer. In order to account for multiple testing, a p value of < 0.01 was considered to be statistically significant (*). Percentages does not add up to 100 due to rounding. The figure includes both patients that received a cancer diagnosis and those freed from cancer suspicion

We wanted to investigate if the pandemic had caused patients to avoid seeking medical help for symptoms that could be caused by cancer. Our data show that respondents sought help in a similar fashion before and during the pandemic (Fig. 1). However, there was a slight increase in the group of patients waiting for the longest period.

**Fig. 2:**
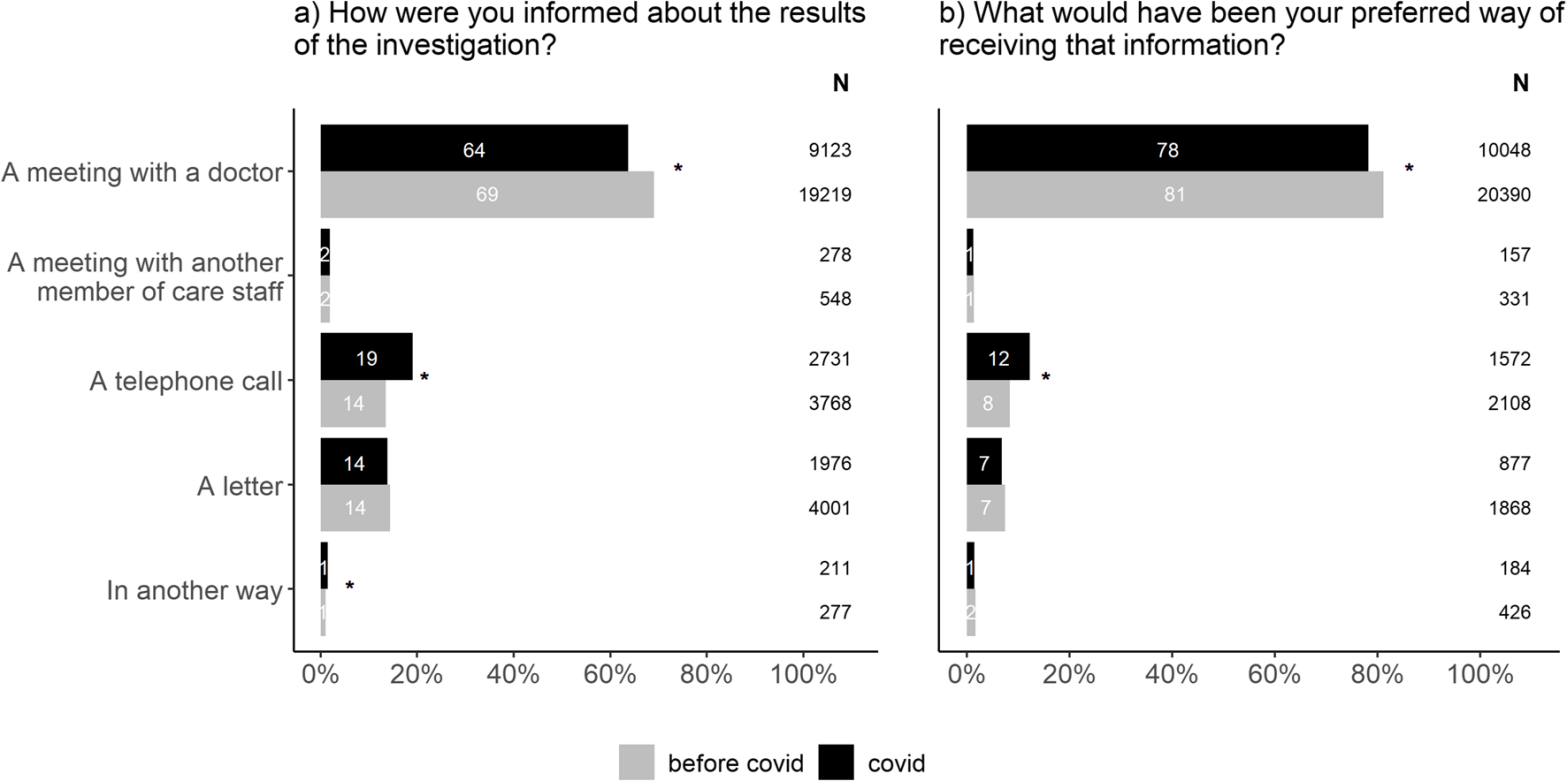
Changes in how patients experienced to be informed about the results of the investigation (**a**) in relation to their preferred way of receiving that information (**b**). The chi-square test was used to compare the proportions between”before covid” and”covid” within each possible answer. In order to account for multiple testing, a p value of < 0.01 was considered to be statistically significant (*). The figure includes both patients that received a cancer diagnosis and those freed from cancer suspicion

As telemedicine has become more common during the pandemic [11], we wanted to investigate if this was the case also for patients investigated for suspected cancer and compare to what extent it matches with the patient’s preference. Our data show that a majority of the patients (64%) still receive the results of the investigation, i.e. a cancer diagnosis or being freed from cancer suspicion, in a meeting with their physician (Fig. 2a). However, the proportion of patients receiving the results of the investigation by telephone increased during the pandemic. The patient preferences follow the same pattern whereby meeting with the doctor is still by far the most preferred alternative, but a larger proportion of patients were open to receiving the results, positive or negative, by telephone during the pandemic (Fig. 2b). Similar patterns could be seen for both patients freed from cancer suspicion and patients that received a cancer diagnosis.

**Fig. 3:**
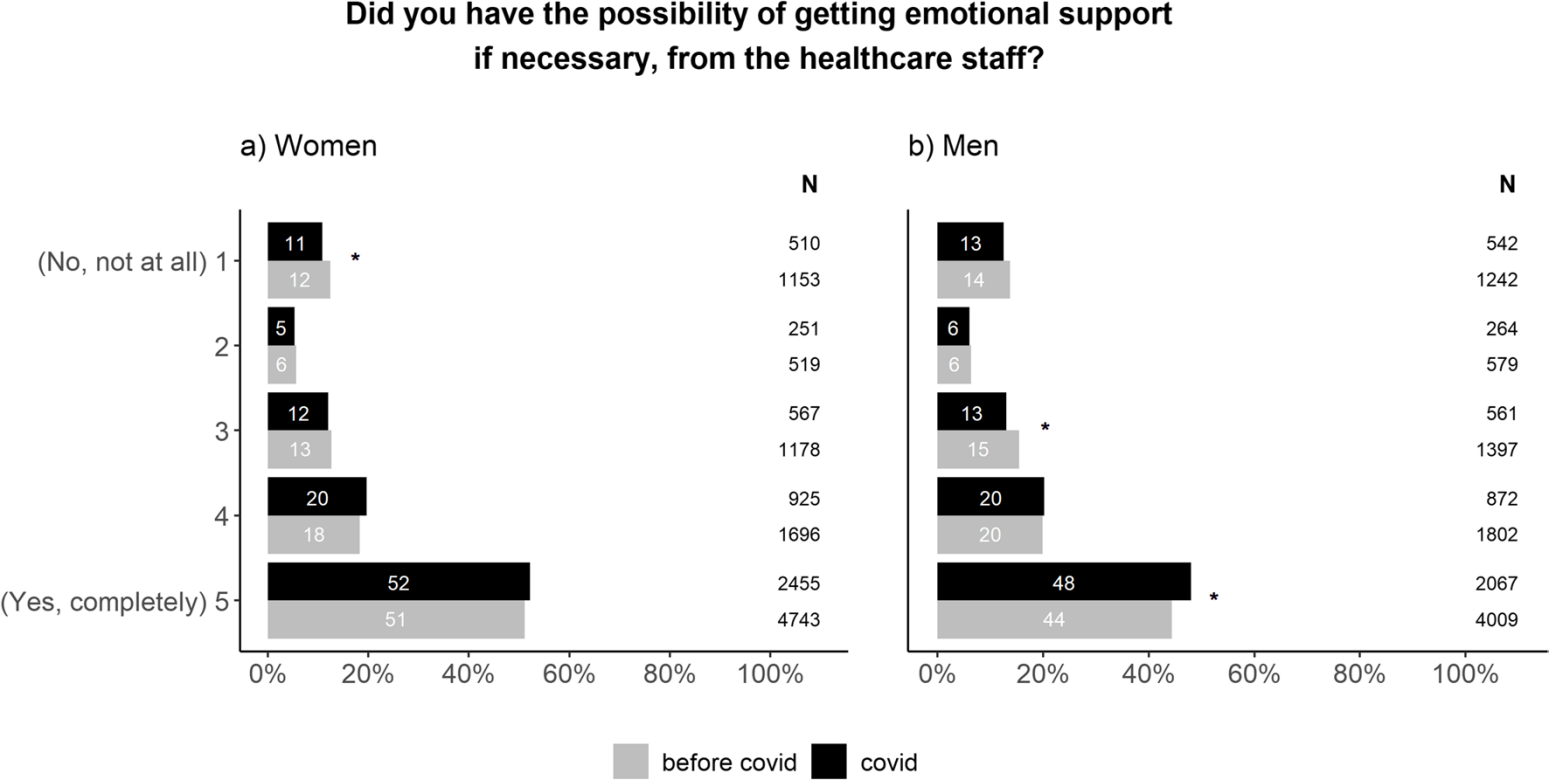
Differences in patient experience of receiving emotional support presented for women (**a**) and men (**b**). The Chi-square test was used to compare the proportions between”before covid” and”covid” within each possible answer. In order to account for multiple testing, a p value of < 0.01 was considered to be statistically significant (*). The figure includes both patients that received a cancer diagnosis and those freed from cancer suspicion

Further, we wanted to investigate if the emotional support offered to patients investigated for cancer and their next of kin had changed during this time period. We found that emotional support to patients had improved during the pandemic compared to before (Fig. 3). The improvement was more prominent in men than in women.

**Fig. 4:**
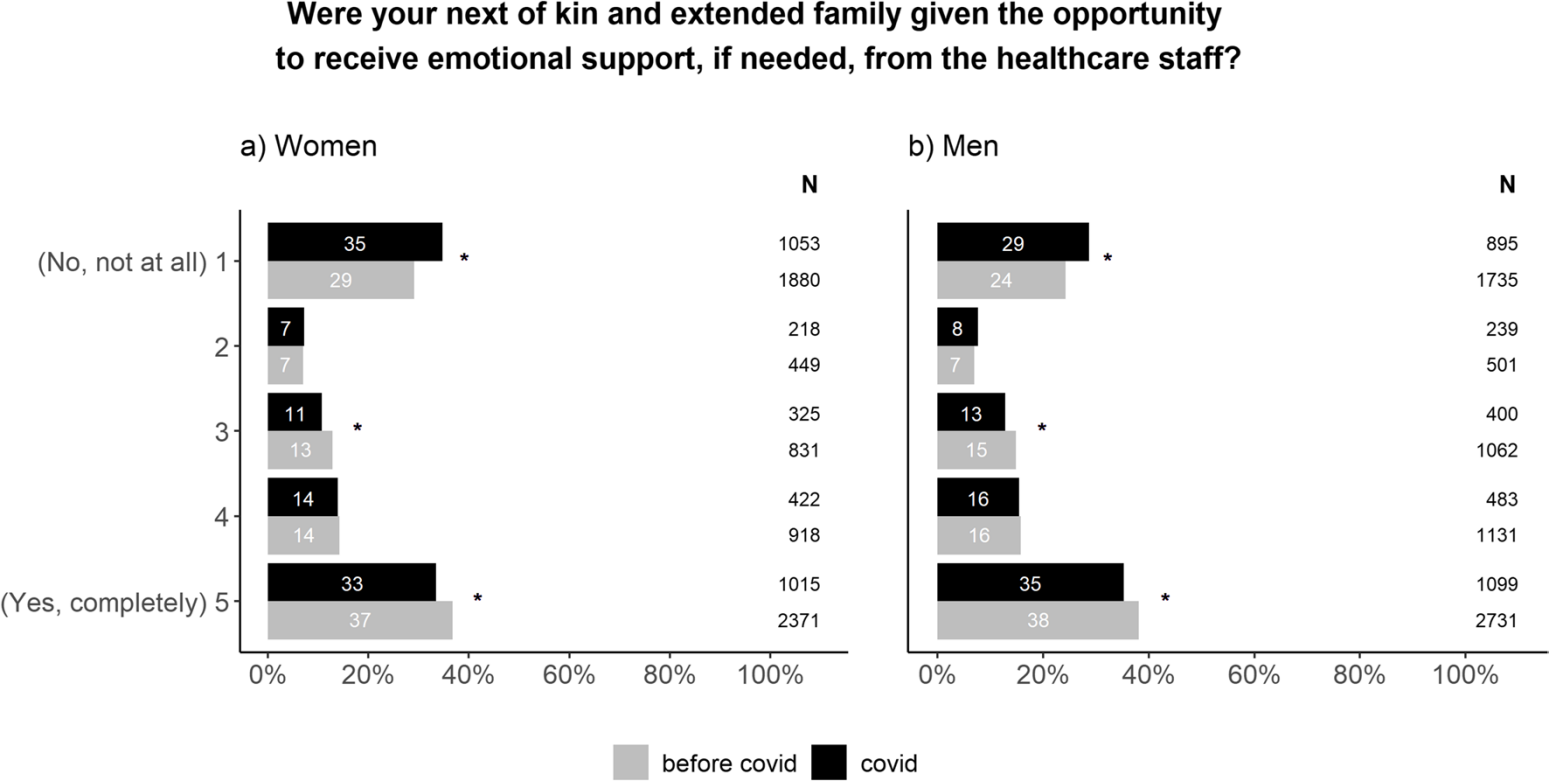
Differences in emotional support to patients’ next of kin and extended family presented for women (**a**) and men (**b**). The chi-square test was used to compare the proportions between”before covid” and”covid” within each possible answer. In order to account for multiple testing, a p value of < 0.01 was considered to be statistically significant (*). The figure includes both patients that received a cancer diagnosis and those freed from cancer suspicion

Emotional support to next of kin, however, declined during the pandemic. The changes were similar for men and women (Fig. 4).

**Fig. 5:**
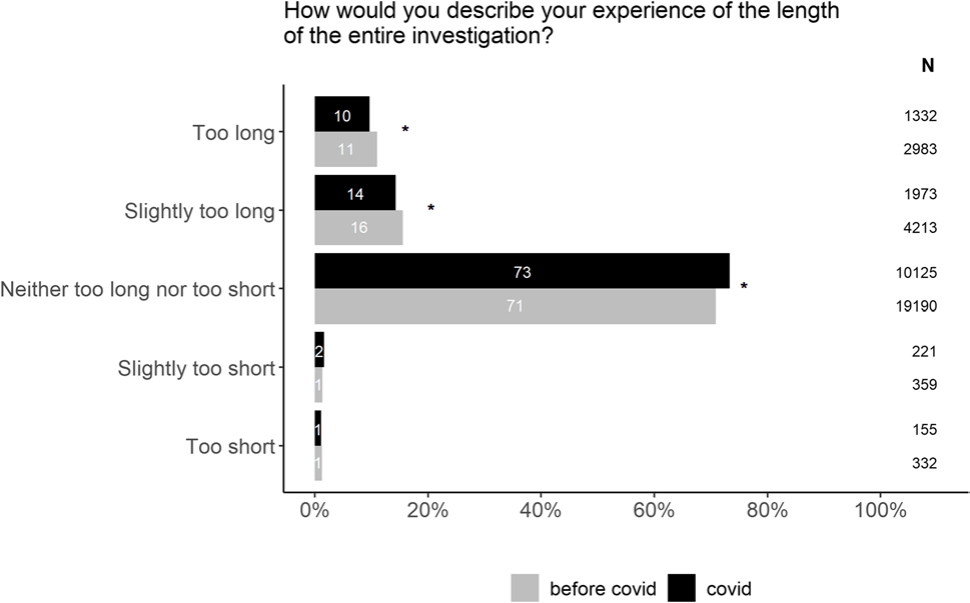
Patients’ experience of the length of the investigation before and during the Covid-19 pandemic. The chi-square test was used to compare the proportions between”before covid” and”covid” within each possible answer. In order to account for multiple testing, a p value of < 0.01 was considered to be statistically significant (*).The figure includes both patients that received a cancer diagnosis and those freed from cancer suspicion

To assess if the experience of the waiting times had changed during the pandemic in patients investigated for suspected cancer, we compared the answers to the question “How would you describe your experience of the length of the entire investigation?” As presented in Fig. 5, the waiting times were perceived to be shorter during the pandemic compared to before.

## Discussion

This study is based on extensive material collected both before and during the Covid-19 pandemic. In our analyses, we have included over 45 000 patients who have answered a questionnaire regarding their experiences of being investigated for cancer.

The answers to many questions in the questionnaire show little or no change during the pandemic compared to before. This indicates resilience within the Swedish health care system as well as in the patient population. Sweden did not opt to go into a full lock-down but remained relatively open [1]. This, in combination with extensive campaigns from the health care system and government to encourage patients to continue to seek medical help for potential symptoms of cancer, could probably explain why respondents report an unchanged pattern for seeking medical help during the pandemic. Further, cancer care was a prioritized area of healthcare in Sweden as well as in other western countries [1]. This meant that fewer resources were reallocated from cancer care compared to other areas. Nevertheless, the total number of cancers diagnosed decreased significantly during the pandemic [5, 6], indicating that many patients refrained from seeking help. A similar drop in the number of cancer cases has also been reported in other countries [15–17]. Patients were conflicted between the instructions of seeking medical assistance early and not bothering health care professionals for minor ailments [18, 19], a feeling that may have increased during the pandemic. Since the survey only was sent to patients that sought help, patients that did not contact the health care system could not be included in the study. This provides a reasonable explanation as to why respondents reported seeking help in the same manner as before while the total number of patients diagnosed with cancer decreased. Fewer patients seeking medical assistance for both cancer and benign conditions has opened possibilities for shorter waiting times for cancer patients in some clinics. Thus, with fewer patients visiting those clinics, more time was available for each patient. This may explain why waiting times were perceived as shorter during the pandemic. This also explain why patients report better emotional support compared to before the pandemic. Although health care staff experienced challenges providing adequate support [20], patients reported an improvement in this area. Both men and women rely primarily on family for support, but it has been suggested that men experience better access to support from medical staff compared to women [21]. It is possible that a change in availability of and contact with healthcare staff during the pandemic benefited men more than women. In general, women also report higher levels of psychological distress during the pandemic [22], which might have influenced the need for support. However, as part of the social distancing strategy, restrictions were applied to the possibilities to bring a next of kin to the hospital [1]. This is the most probable explanation as to why the emotional support to significant others has deteriorated. To our knowledge, there are no publications on proxy-reports of emotional support to significant others. It has been suggested that female partners to cancer patients experience more distress than male partners [23]. In our study, the pandemic resulted in a similar decline in emotional support in both men and women. Significant others play an important role in the care for cancer patients as they help with daily care and have extensive knowledge about the patient. They also provide the possibility for health care staff to quickly respond to changes in patient condition, thus creating resilience in health care [24]. Providing them with emotional support when needed is therefore necessary.

While most patients still prefer to be informed about the results of the investigation in a meeting with the doctor, an increasing number of patients have opened up to receiving the results by telephone. This shift in interest in favor of technical solutions is in line with previous work [11, 25]. Before the pandemic, only 8% of patients listed this alternative as their preferred way, while 12% preferred telephone to meeting with the doctor during the pandemic. Interestingly, this shift can be seen for both patients diagnosed with cancer and patients being cleared from cancer suspicion. This opens up for a new approach to patient meetings. While some meetings need to remain as physical meetings, others could be converted to a digital format [10, 11]. It is reasonable to assume that the particular meeting where a patient receives a cancer diagnosis is not particularly well suited for a conversion to a telephone or video call. However, the shift in patient preference even for this meeting should be taken seriously as technical solutions may be beneficial for both patients and health care professionals. As long as the patient’s preferences are respected, bad news can be delivered by telephone without causing negative psychosocial consequences for the patient [26]. During the pandemic, the question of how to best deliver such news with few available alternatives came into focus [27]. Upholding the ability for patients to feel welcomed, seen and listened to by health care professionals is crucial for a good patient experience [28].

In our material there were differences between patients that ended the investigation with a cancer diagnosis and those that were freed from cancer suspicion. However, these differences were present before the pandemic and thus unrelated to Covid-19. They are most probably related to other factors outside the scope of this study, such as differences in subsequent care for the patients with cancer and patients freed from cancer suspicion. Moreover, this demands a more detailed analysis to understand the reasons behind these differences and the potential impact of these differences. We intend to address this in future work.

### Study limitations

Questionnaire-based studies have a responder bias. Individuals with strong opinions are more likely to participate in this kind of survey. Also, national as well as international data show that the total number of cancers diagnosed has dropped significantly during the pandemic. Our survey only reaches patients that come into the health care system. Those who chose not to seek medical help, out of fear for Covid-19 or for other reasons, could not be included. Our data from the Covid-19 period may thus represent those less prone to change which in turn may cause us to underestimate the effect of the pandemic throughout the study.

Given the size of the material, even small changes can be detected and appear statistically significant. Therefore, clinical relevance needs to be assessed on hospital or diagnosis basis.

### Conclusion

Swedish cancer care showed resilience during a time of extraordinary circumstances and unprecedented pressure. Patients continuing to seek medical help for symptoms related to cancer perceived the waiting times as shorter during the pandemic and adaptations were made to meet the needs of patients on several levels. The emotional support to patients has functioned well, whereas the support to next of kin has deteriorated.

## Data Availability

Data is publicly available in restricted form at https://resultat.patientenkat.se/ (in Swedish). Deidentified participant data that underlie the results of this article will be made available from the authors, upon reasonable scientific request and approved ethical board application. Requests for data will be reviewed on a case-by-case basis.
